# Physio-pathological effects of N6-methyladenosine and its therapeutic implications in leukemia

**DOI:** 10.1186/s40364-022-00410-3

**Published:** 2022-08-23

**Authors:** Wei-Wei Liu, Hao Wang, Xiao-Yu Zhu

**Affiliations:** 1 School of basic medical sciences, Shandong University, Jinan, China; 2 Department of Clinical Laboratory, The First Affiliated Hospital of USTC, Division of Life Sciences and Medicine, University of Science and Technology of China, Hefei, China; 3 Department of Hematology, the First Affiliated Hospital of USTC, Division of Life Sciences and Medicine, University of Science and Technology of China, Hefei, China; 4 Blood and Cell Therapy Institute, Division of Life Sciences and Medicine, University of Science and Technology of China, Hefei, China; 5 Anhui Provincial Key Laboratory of Blood Research and Applications, Hefei, China

**Keywords:** N6-methyladenosine, Hematopoietic stem cells, Hematopoiesis, Leukemia

## Abstract

*N*6-methyladenosine (m6A), the most prevalent epigenetic modification of RNA in mammals, has become a hot topic throughout recent years. m6A is involved with every links of the RNA fate, including RNA splicing, nuclear export, translation and stability. Due to the reversible and dynamic regulatory network composed of ‘writers’ (methylase), ‘erasers’ (demethylase) and ‘readers’ (m6A binding proteins), m6A has been deemed as an essential modulator in vast physiological and pathological processes. Previous studies have shown that aberrant expression and dysfunction of these regulators are implicated in diverse tumors, exemplified by hematological malignancies. However, we should hold a dialectic perspective towards the influence of m6A modification on leukemogenesis. Given that m6A itself is neither pro-oncogenic nor anti-oncogenic, whether the modifications promote hematological homeostasis or malignancies occurrence and progression is dependent on the specific targets it regulates. Ample evidence supports the role of m6A in maintaining normal hematopoiesis and leukemogenesis, thereby highlighting the therapeutic potential of intervention in m6A modification process for battling leukemia. In this review, we introduce the advances of m6A modification and summarize the biological functions of m6A in RNA metabolism. Then we discuss the significance of several well-studied m6A regulators in modulating normal and malignant hematopoiesis, with focus on the therapeutic potentials of targeting these regulators for battling hematopoietic malignancies.

## Introduction

Hematopoietic homeostasis is a delicately orchestrated balance between the proliferation and differentiation of hematopoietic stem/progenitor cells (HSCs) [1]. Accumulated genetic and epigenetic abnormities will deflect HSCs’ trajectory and convert HSCs into leukemia stem cells (LSCs), leading to malignant expansion of poorly differentiated cells [2]. Among all those hematological malignancies, acute myeloid leukemia (AML) is the most well-learned one, characterized by high lethality, unfavorable prognosis, frequent relapse and chemotherapeutic resistance [3]. Numerous factors can drive the occurrence and development of AML, among which epigenetics has been underlined by increasing evidence. Generally speaking, epigenetics centers on heritable variations in gene expression or cell phenotype without changing the nucleotide sequence. It can be classified as transcriptional, posttranscriptional and posttranslational, depending on the levels of modifications. Mounting evidences showed an important determinant of epigenetic abnormalities in the initiation and progression of hematological malignances, emphasizing the potential of novel epigenetic therapies [4, 5]. Until now, therapeutics targeting DNA methyltransferase and histone deacetylase have achieved remarkable effects against AML [4]. In addition to the well-studied DNA methylation and histone modification, RNA modifications exert modulatory roles at the post-transcriptional level.

For the past few years, the implication of RNA modification in AML have refueled the scientific interest in developing new therapeutic strategies. Among over 170 kinds of RNA modifications [6], N6-methyladenosine (m6A), which refers to methylation at the N6 position of adenosine, is identified as the most prevalent and abundant one [7]. Owing to the development of high-throughput sequencing, several cutting-edge m6A identification method has emerged, such as m6A-seq and miCLIP-seq, which gradually shape the cellular m6A landscape [7–9]. m6A marks exist in approximately 1/3 of the mRNAs, while a majority of these hold only one m6A mark, only a minority harbor more than 20 [10]. With our perception of regulatory machinery improving, m6A methylation is verified to play a crucial role in RNA fate, including splicing, nuclear export, translation, stabilization, and phase separation. Notably, the m6A modifications are not exclusive for mRNA, but also found in many other RNAs, including transfer RNA (tRNA), ribosomal RNA (rRNA), and long noncoding RNA (lncRNA) etc. Continuous evidences indicate that m6A methylation is widely involved in fate of other RNAs, such as the expression and stabilization of lncRNAs [11, 12], processing of pri-miRNA and maturation of miRNA [13], metabolism of circRNA [14]. Regulating gene expression via methylation and demethylation, m6A participates in numerous biological and pathological processes, including multiple human malignancies, adipogenesis and type2 diabetes [15–17].

In this review, we summarize the established knowledge on m6A methylation in mRNA metabolism, then describe the mechanisms and biofunctions of several crucial m6A regulators in normal hematopoiesis and leukemogenesis, particularly focusing on their potential value as targets for clinical therapy.

### Regulation of m6A RNA modification

#### Writers (methyltransferases)

The functions in cellular m6A deposition are achieved by a multicomponent methyltransferase complex (MTC), a heterodimer composed of METTL3 and METTL14 [18], as well as the regulatory subunits WTAP [19], RBM15 [20], VIRMA [21], and ZC3H13. METTL3, the first identified writer [22], is the catalytic subunit of MTC. It is composed of nuclear localization signal (NLS) domain, leading helix structure (LH) domain, CCH-type zinc finger domain (ZFD), and two methyltransferase domains (MTD). NLS and LH mediate the interaction between METTL3 and METTL4, MTD binds to the methyl donor SAM and ZFD is responsible for recognizing targets [23, 24]. The target sites of METTL3 are enriched in the motif RRACH (R = G or A, H = A, C, or U) in the 3' untranslated region (3' UTR), in the long internal exon, and near the stop codon in mRNA [7]. As for METTL14, its crucial role in facilitating RNA substrate binding has been widely accepted [23]. And it can be promoted by PRMT1 via methylating its C-terminus [25]. WTAP is responsible for the localization of METTL3/METTL14 into nuclear speckles and recruiting them to targets. Meanwhile, its function and homeostasis are under the regulation of the complex [19, 26]. RBM15, along with its paralog RBM15B, recruits MTC to specific sites of mRNA and lncRNA X-inactive specific transcript (XIST) [20, 27]. VIRMA, the largest constituent of methyltransferase indispensable for the entire methylation process [28], recruits METTL3/METTL14/WTAP to selectively deposit in the 3' UTR and near the stop codon [21, 29]. ZC3H13 plays a vital regulatory role by anchoring the complex in the nucleus, the loss of which will initiate export of nuclear WTAP followed by lack of METTL3/METTL14 [30, 31]. METTL16 has been recognized as a conserved U6 snRNA methyltransferase with a comparatively limited scope of targets with a conserved UACAGAGAA sequence, independent of MTC [32–34]. The N-terminal domain exerts the enzymatic activity, while the C-terminal is composed of two vertebrate-conserved regions (VCRs) [35, 36]. METTL16 was verified to bind to hairpin in the 3' UTR of MAT2A, participating in the homeostasis of SAM by regulating the stability of the SAM synthetase in a m6A-dependent manner [34]. Recently, METTL16 has been found to exert both methyltransferase activity-dependent and -independent functions, similar to METTL3 [37]. Moreover, some studies have revealed the mechanisms of m6A modifications on other RNAs. For instance, ZCCHC4 is found to modify 28 S ribosomal RNA [38, 39], and METTL5/TRMT112 complex is supposed to correlate with 18 S rRNA [40].

#### Erasers (demethylases)

Erasers are a group of regulators that remove m6A deposition, mainly including the Fe2 + and alpha-ketoglutarate (a-KG)-dependent AlkB family members FTO and ALKBH5 [41, 42]. In particular, the discovery of FTO indicates the m6A modification is a reversible process, representing a milestone in the research of m6A. Although FTO and ALKBH5 are homologues, it has been observed that they have different substrate affinity, due to their distinct active-site residues [43]. In contrast to ALKBH5, which exclusively demethylates m6A, the principal substrate of FTO remains controversial [44, 45]. Mauer et al. hold that FTO’s primary target was not m6A but N6,2'-O-dimethyladenosine (m6Am), since m6A start to be demethylated by FTO at a concentration twice of m6Am in vitro [44, 46]. But Zhang et al. has discovered FTO exhibits equivalent catalytic activity toward m6A and m6Am after displaying on the same RNA sequence [47]. In vivo, according to the study conducted by Wei et al., FTO regulate m6A in nucleus, but this affinity bias tends to be inconspicuous in cytoplasm, which is ascribed to the abundance of m6A in nuleus [45]. Furthermore, m6A in mammalian transfer RNA (tRNA) has been found as a novel substrate of ALKBH3, and demethylation can significantly enhance the translation efficiency in vitro [48].

#### Readers

Upon recognizing and binding to specific m6A sites, readers mediate the distinct functions in regulating RNA fate and gene expression [49]. YT521-B homologue (YTH) protein family, as the first group of readers, is composed of YTHDF1/2/3 and YTHDC1/2. Among them, only YTHDC1 localizes to the nucleus while the rest in cytoplasm [50, 51]. With YTH domain directly binding to m6A marks, they are extensively involved in RNA metabolism [52–54]. DF proteins are found to bind to CNOT1 and mediate the deadenylation of mRNA [55]. And it was revealed that YTHDC2 can function as helicase independent of m6A, enhanced by its interaction with the 5’?3’ exoribonuclease XRN1 [56]. Contrary to the prevailing opinions, Zaccara et al. revealed that DF proteins bound the same m6A-marked mRNAs rather than different ones and the DF paralogs act redundantly in mediating mRNA degradation and cellular differentiation. Furthermore, they proposed a unified model that all m6A-marked mRNAs were subjected to the combined action of YTHDF proteins, which was proportional to the number of m6A sites [57]. The second group, the heterogeneous nuclear ribonucleoprotein (HNRNP) family, includes HNRNPC, HNRNPG, and HNRNPA2B1. Among them, HNRNPC mediates pre-mRNA processing through ‘the m6A-switch’ mechanism, wherein m6A alters the RNA structure to facilitate the binding [58]. Katherine and co-workers revealed an integrated mechanism of co-transcriptional m6A-mediated splicing regulation in which HNRNPG directly interacted with RNA polymerase II [59]. The third member in this family, termed as HNRNPA2B1, functions through directly binding primary miRNA to elicit alternative splicing effect. Meanwhile, its interaction with the microRNA Microprocessor complex protein DGCR8 promoted primary miRNA processing [60]. The third group of readers is the insulin-like growth factor 2 mRNA-binding protein family, IGF2BP1/2/3, possessing 4 repetitive KH domains. IGF2BPs specifically bind to m6A-marked RNA with KH3/4, enhancing the mRNA stability and facilitate the translation [61, 62]. Though the regulatory mechanism remains unraveled, previous study proved that YBX1 cooperates with IGF2BPs to stabilize m6A-tagged RNA [62]. Aside from the above three groups, novel readers are continuously being discovered. For example, PRRC2A (Proline rich coiled-coil 2A) is found to play an important role in oligodendrocyte specification through binding to m6A site on Olig2 mRNA [63].

### Functions of m6A in mRNA fate

**Fig. 1:**
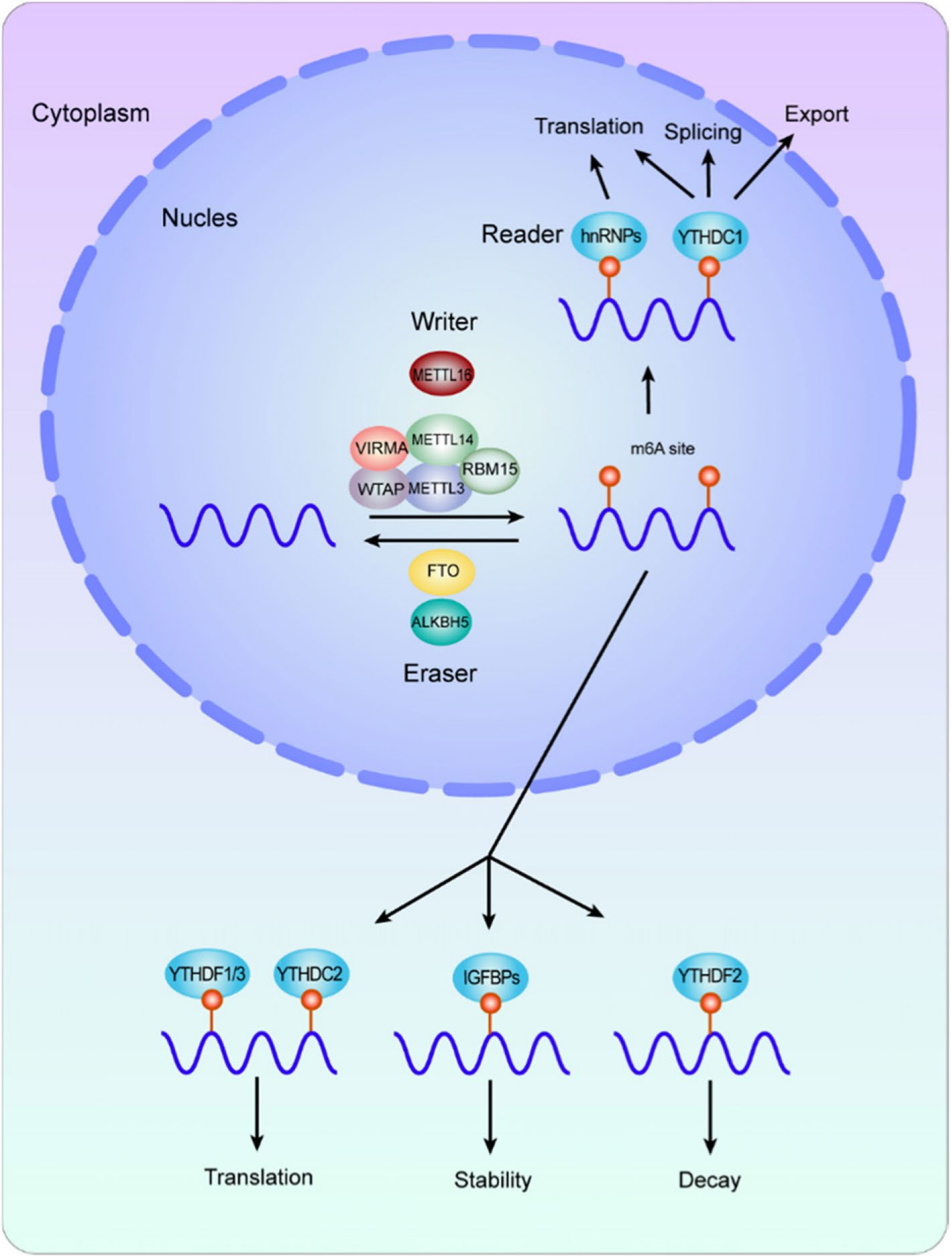
Molecular composition and regulation mechanism of m6A methylation modification. m6A methylation is a dynamic and reversible process coordinated by methyltransferases (defined as “writers”, including METTL3, METTL14, WTAP, ZC3H13, HAKAI, VIRMA, and RBM15), demethylases (defined as “erasers”, FTO and ALKBH5), and “readers”, such as YTHDF1-3, YTHDC1, IGF2BPs, HNRNPC, HNRNPA2B1, and eIF3. These molecules recognize and bind to m6A-modified RNA and thus mediate RNA splicing, stability, translation, and RNA nuclear export

As mentioned above, writers and erasers act in methylation and demethylation, respectively. Readers preferentially recognize m6A modifications, and thus determine the fate of the methylated RNAs, including RNA splicing, exportation, translation, and stability (Fig. 1). For pre-mRNA splicing, the interaction between m6A regulators and splicing factors could affect the alternative splicing pattern, leading to exon skipping and abnormal transcripts. For nuclear export, several regulators have been verified to play a significant role. For translation, the m6A-mediated regulation is intricate and multi-layered, including direct and indirect mechanisms. As for mRNA stability, whether maintaining stability or promoting degradation depends on the readers, via specific pathways. And m6A modifications can enhance phase separation, which further determine the fate of RNAs.

#### Pre-mRNA splicing

Splicing is a critical step in post-transcriptional regulation, in which the alternatively removing of introns and joining exons produce diverse transcripts. m6A modification sites are spatially overlapping with mRNA splicing enhancer binding regions, thus regulate mRNA alternative splicing [17]. Precious studies have found that downregulation of m6A writers interferes splicing and gene expression [64, 65]. As noted, METTL16-dependent m6A in the U6 spliceosomal snRNA and 3'-UTR of mRNA impacts RNA splicing and stability [36, 66]. Meanwhile, demethylases also show significant influences in regulating splicing. As the preferential binding sites of FTO are adjacent to the alternative splicing exon and polyA site, the demethylation tends to inhibit recruitment of serine/arginine-rich splicing factor 2 (SRSF2) and induce exon 6 skipping [17, 67]. Also, suppression of ALKBH5 results in exon jumping in transcripts, which are rapidly degraded [68]. Additionally, ALKBH5 exerts its regulatory effect by promoting the phosphorylation of ASF/SF2, the hyperphosphorylated form of which is involved in splicing [41]. As mentioned before, hnRNPA2B1 gets access to target RNA via an “m6A switch” mechanism, then regulate RNA splicing in a METTL3-dependent way. Furthermore, loss of hnRNPC/hnRNPG will alter the splicing pattern [58–60]. Wen et al. identified that YTHDC1 modulates pre-mRNAs splicing through facilitating SRSF3 and repelling SRSF10 [69].

#### RNA nuclear export

After being fully processed, mRNAs are translocated into cytoplasm, where writers, erasers, readers all participate in [70]. The interaction between YTHDC1 and SRSF3 facilitates the export of RNA via modulating the contact between RNA and nuclear RNA export factor 1 (NXF1) [53]. FMRP, as a reader, is indispensable in the nuclear export mediated by Exportin 1 (XPO1) [71, 72]. ALKBH5 is involved in phosphorylation of ASF/SF2 and the hypophosphorylated ASF/SF2 facilitates mRNA export by mediating the interaction of the TAP-p15 complex and mRNA [73, 74], thus ALKBH5 is responsible for determining the subcellular location of mRNAs. Consistent with that, polyA RNA accumulation in the cytoplasm was observed in ALKBH5-deficient cells, attributed to the augmented nuclear RNA export [41].

#### RNA translation

m6A-related translation regulation plays a pivotal role in several cancer and some normal physiological processes. METTL3 can regulate translation in both methyltransferase catalytic -dependent and -independent manner. The former pattern is more common, in which METTL3 methylated mRNA and then readers mediated the specific regulation [75]. METTL3 also has impact on the biogenesis of ribosome via modulating the PES1 expression [75]. As for the indirect regulatory mechanism, METTL3 interacts with eIF3h to enhance translation and generates densely packed polyribosomes [76, 77], which is distinguished from the stress-inducing mechanism [78]. Similarly, Rui Su et al. discovered that METTL16 could promote translation by directly interacting with the eIF3a, eIF3b and rRNA, thereby facilitating the assembly of the translation-initiation complex in the cytosol [37]. It has been verified that the interaction of YTHDFs with translation machinery can enhance translation. Mechanistically, YTHDF1 facilitates the cap-dependent translation initiation by forming a loop structure with eIF4G and eIF3, consequently recruiting ribosomes [79]. In addition to collaborating with these translational factors, YTHDF1 could advance the expression level of eIF3C in an m6A-dependent manner [80]. Similarly, YTHDF1 knockdown leads to significant decrease of eIF3A and 3B in Merkel Cell Carcinoma [81]. Although eIF4 complex is necessary in typical translational initiation, YTHDF1 could provoke initiation in the absence of eIF4 complex, through eIF3A and 3B [81]. Zhou et al. observed that m6A abundance induced by heat shock conditions in 5'-UTR could promotes the initiation of cap-independent translation [78]. Later, Liu et al. revealed that the stress-induced m6A modification in the 5'-UTR promotes mRNA translation via binding to eIF3 independent of YTHDF1 [80]. Synergizing with YTHDF1, YTHDF3 promotes translation by interacting with 40 and 60 S ribosome subunits [52, 82]. YTHDC2, with RNA helicase activity, boosts translation efficiency but declines mRNA abundance [83, 84].

#### RNA stability

The mRNA stability governs the equilibrium of RNA metabolism and gene expression. While some studies support the negative correlation between m6A modification and mRNA stability [28, 85], existing experimental results indicating that m6A modification serves as a double-edged sword in regulating mRNA stability [61, 86, 87]. As a well-established regulator of RNA decomposition, YTHDF2 selectively binds to target mRNA via its C-terminal domain, and the *N*-terminal domain is responsible for localizing the YTHDF2-mRNA complex to RNA decay site, where the deadenylase complex CCR4-NOT was recruited [55, 88]. FTO can increase the stability of MYC mRNA by inhibiting the m6A-dependent RNA decay mediated by YTHDF2 [89]. YTHDF3 is in synergism with YTHDF2 to induce mRNA degradation [90]. And YTHDF1 was found to induce the degradation of MAT2A mRNA by recognizing its 3’-UTR m6A site [34]. In contrast to YTHDF2, readers IGF2BP1/2/3 could improve mRNA stability with its KH domain binding to m6A site under normal and stress conditions [61]. Additionally, FMRP and PRRC2A have also been proven to enhance mRNA stability in an m6A-dependent manner [86, 87]. Thus, some oncogenes with decreased m6A level were observed to be less stable.

#### Phase separation

Phase separation, especially liquid–liquid phase separation (LLPS), drives the formation of compartmentalization in a membraneless manner. It has been highly considered as a significant strategy for various biological reactions [91]. Since m6A can alter the charge, conformation and protein anchoring of modified RNAs, it can further regulate phase separation by influencing RNA-protein and RNA-RNA interactions. Several studies have verified that YTHDFs could lead to phase separation both in vivo and in vitro, closely correlated with the formation of stress granules, P granules and neuronal RNA granules [92–94]. The LCDs of YTHDFs can trigger phase separation without RNAs in vitro, but the direct binding between YTH domains and m6A sites is required for phase separation in vivo [92]. Recently, Jiong Li et al. revealed that YTHDF1 interacts with AGO2 to promote mRNA degradation through LLPS [95]. Also, the functions of YTHDC1 in bioprocesses are closely correlated with phase separation. YTHDC1 is reported to mediate chromatin remodeling via phase separation [96]. In mechanism, it promotes the demethylation of histone mark H3K9me2 by recruiting demethylase KDM3B [97]. YTHDC1 can recognize the m6A sites on lncRNA XIST and further recruit repressive proteins to induce gene silencing, which is supposed to be mediated by phase separation [20, 98]. Similarly, the function of YTHDC1 in alternative splicing is supposed to be modulated by phase separation [99].

### Role of m6A in hematopoietic malignancies

**Fig. 2:**
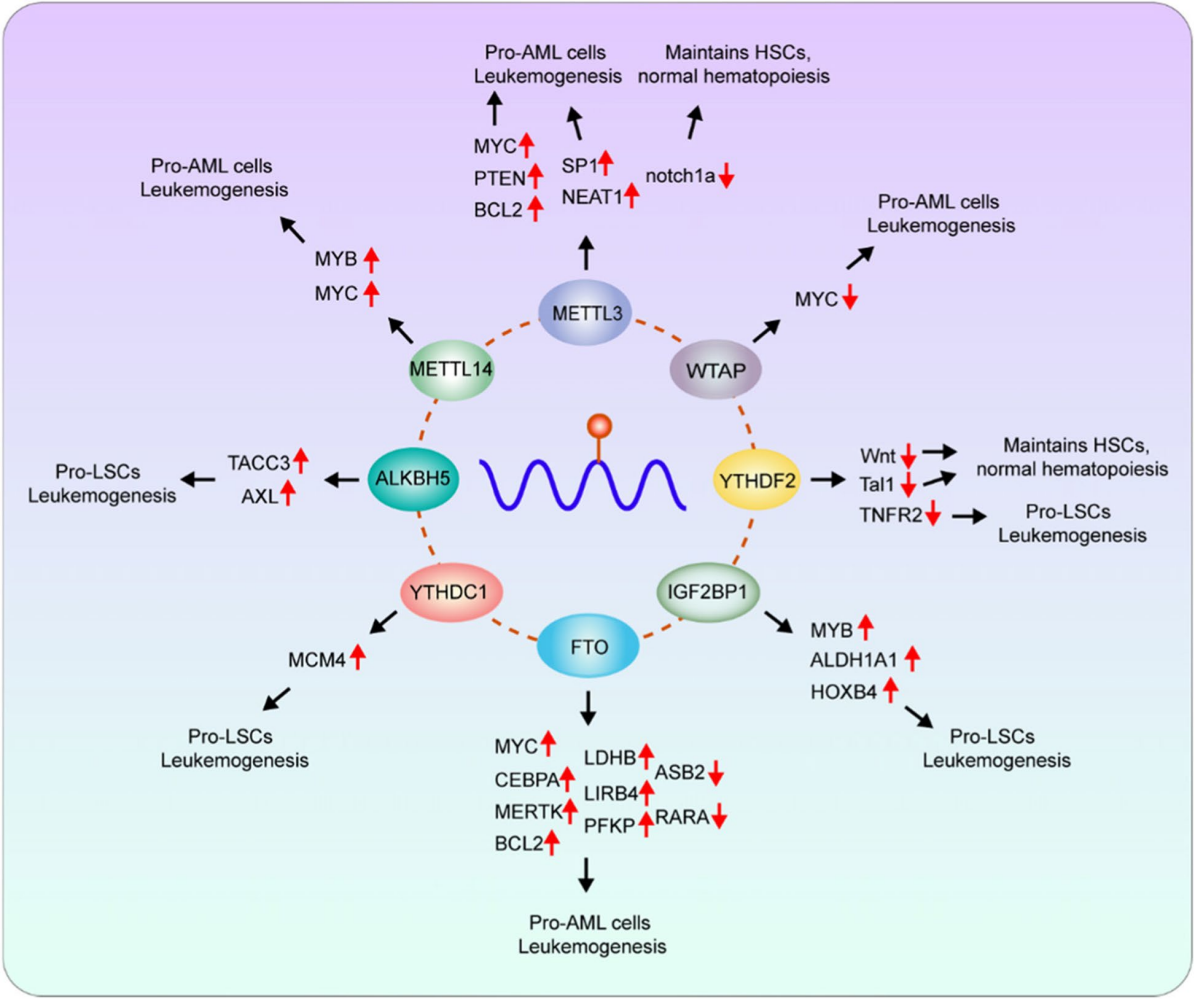
Targets and molecular mechanisms of m6A regulators

Normal hematopoiesis hinges on the balance between regeneration and differentiation of HSCs, which is under rigorous and elaborate control. Dysregulated HSCs will transform into LSCs, accounting for the occurrence of hematopoietic malignancies, as well as the poor prognosis and chemotherapeutic resistance. The m6A methylation is supposed to participate in almost every crucial process, and some underlying mechanisms have been illuminated (Table 1; Fig. 2). Importantly, previous studies revealed the therapeutic potential of several regulators. Small-molecule inhibitors of METTL3, FTO, ALKBH5 have recently been identified and have exhibited considerable anti-leukemia effects both in vitro and in mouse models (Table 2).

**Table 1:** The target genes, biological functions and mechanisms of RNA m6A methyltransferases, demethylases, and binding proteins in hematopoietic malignancies

Regulator	Target genes	Mechanisms	Biological functions	Reference
METTL3	MYC, BCL2, PTEN	Promotes translation of MYC, BCL2, PTEN and inhibits pAKT pathway	Promotes cell proliferation and colony formation, inhibits differentiation and apoptosis	[100]
	SP1	Promotes translation of SP1	Promotes cell proliferation and inhibits differentiation	[101]
	notch1a	decreases stability of notch1a mRNA and inhibits Noctch signaling pathway	Promotes HSCs generation through the endothelial-to-hematopoietic transition (EHT)	[102]
	No study	No study	Maintains HSCs in a quiescent state	[103]
METTL14	MYB, MYC	Increases mRNA stability and promotes translation of MYB, MYC	Promotes cell proliferation and colony formation, inhibits differentiation and apoptosis	[104]
WTAP	MYC	Decreases mRNA stability	Promotes cell proliferation, colony formation and chemoresistance, inhibits differentiation	[105]
	No study	Hsp90 maintains the protein stability of WTAP	Promotes cell proliferation, colony formation and chemoresistance, inhibits differentiation	[106]
FTO	MYC, CEBPA	Increases mRNA stability	Promotes cell proliferation	[89]
	ASB2, RARA	Decreases mRNA stability	Promotes cell proliferation, colony formation and chemoresistance, inhibits differentiation and apoptosis	[107]
	PFKP, LDHB	Increases mRNA stability	Promotes aerobic glycolysis	[108]
	MERTK, BCL2	Increases mRNA stability	Promotes cell proliferation and drug resistance	[109]
	LILRB4	Increases mRNA stability	Promotes decitabine-induced immune evasion and makes AML cells more resistant to T cell cytotoxicity	[110]
ALKBH5	AXL	Increases mRNA stability	Promotes cell proliferation and colony formation, inhibits differentiation and apoptosis	[111]
	TACC3	Increases mRNA stability	Promotes cell proliferation and colony formation, inhibits apotosis	[112]
YTHDF2	TNFR2	Decreases mRNA stability	Promotes cell proliferation and inhibits apotosis	[113]
	Wnt target genes	Promotes mRNAs degradation of Wnt target genes	1s in a quiescent state	[103]
	Tal1	Promotes mRNAs degradation of key transcription factors	Maintains HSCs in a quiescent state	[114]
YTHDC1	MCM4	Promotes DNA replication	Promotes cell proliferation and inhibits apotosis	[115]
IGF2BP1 IGF2B3	ALDH1A1, HOXB4, MYB RCC2	Increases the expression Increases mRNA stability	Promotes cell proliferation, colony formation and chemoresistance, inhibits differentiation Promotes cell proliferation and inhibits apotosis	[116, 117]
RBM15	Notch pathway	promotes Notch-induced HES1 transcription	Inhibits differentiation	[118]

#### METTL3

**Fig. 3:**
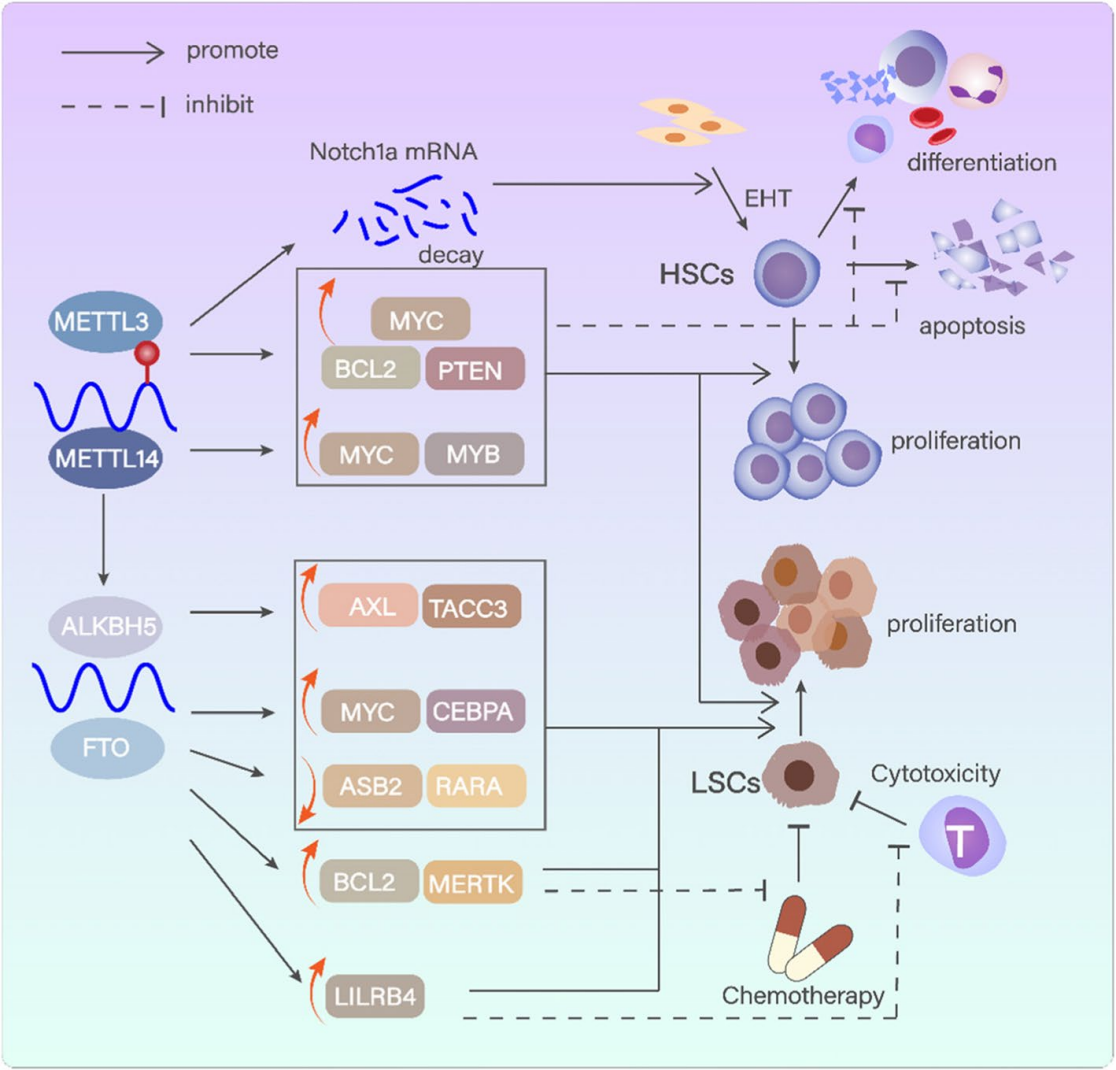
m6A methyltransferases and demethylases in modulating normal and malignant hematopoiesis

Given its vital role in maintaining the normal functions and quiescent state of HSCs, the depletion of METTL3 promotes HSCs differentiation and reduces cell proliferation (Fig. 3). On the contrary, METTL3 overexpression prohibits cell differentiation and triggers cell growth [100]. Via increasing the m6A level of MYC, BCL2 and PTEN, METTL3 facilitates their translation, thereby leading to augmented proliferation and reduced differentiation of AML cells [84]. Meanwhile, it was proved that overexpression of MYC could rescue the deficiency of METTL3 by maintaining symmetrical differentiation [119]. METTL3 associates with chromatin and migrates to transcriptional start site (TSS) of active genes, where CAATT-box binding protein CEBPZ was located. Promoter bound METTL3 initiates the m6A modification of the target mRNA, and subsequently induces the translation by relieving ribosome stagnation [101]. Moreover, METTL3 is supposed to regulate HSC generation via affecting endothelial-to-hematopoietic transition (EHT). Loss of METTL3 delays the YTHDF2-mediated degradation of Notch1a, thus activating Notch signaling pathway which hinders the transition of endothelium into HSCs [102]. In normal hematopoiesis, the expression of METTL3 was alleviated in mature differentiated myeloid cells on a regular basis. The abundance of METTL3 contributes to leukemogenesis and maintenance of the malignant proliferation and undifferentiated state of LSC. Besides, METTL3 has been validated to promote chemoresistance against AML. The downregulation of METTL3 in AML-MSCs upregulates AKT expression, enhances adipogenesis, and results in chemoresistance [120]. MEG3 could elevate expression of miR-493-5p which targeted METTL3/MYC axis, thus promotes the chemosensitivity of AML cells [121].

In addition to causal roles in AML, emerging studies have correlate METTL3 with the initiation and progression of ALL and CML. Ianniello et al. demonstrated that upregulated METTL3/METTL14 could drive the malignant proliferation of CML cells and mediate the resistance against TKIs [122]. METTL3 concurrent with facilitating chemoresistance and restraining autophagy via lowering PTEN mRNA stability in a YTHDF2-dependent manner, METTL3 also controls the progression of CML through m6A modification of lncRNA and the consequent modulation of miR-766-5p/CDKN1A axis [123]. The enrichment of METTL3 was observed in ETV6/RUNX1-positive ALL, while comparatively less expressed in recurrent patients [124, 125]. Besides, Liu and co-workers have unraveled the positive correlation between METTL3 gene polymorphism and the risk of pediatric ALL [126].

The therapeutic potential of METTL3 as an anti-leukemia target has elicited considerable interest, for exploring its inhibitors to intervene the proliferation, differentiation, and apoptosis of leukemia cells. Increasing researches revealed that METTL3 inhibitor treatment results in dose-dependent reduction in m6A level in several cell lines, without alteration in other RNA modifications [127]. Tzelepis et al. have discovered two selective inhibitors of METTL3, which dramatically decreased the expression of METTL3-mediated m6A proteins at the nanomolar level, such as SP1. And they remarkably suppressed tumor growth of in AML model. Throughout METTL3 inhibitors developed the most recently, STM2457 was testified to prolong survival in different mouse AML models [128]. Built upon the structural optimization of a series of METTL3 inhibitors, UZH2 was identified to potently inhibit the function of METTL3 [129].

#### METTL14

As a component of MTC, the expression pattern of METTL14 is similar to METTL3, which is enriched in HSCs (Fig. 3). A recent study displayed that in normal CD34 + HSCs, deficiency in METTL14 promotes differentiation, decreases colony formation, but has no obvious effects on cell growth and apoptosis [104]. Particularly, METTL14, negatively regulated by SPI1, plays a critical role in normal hematopoiesis by facilitating the expression of two oncogenic transcription factors, MYB and MYC [104]. Thus, METTL14 is indispensable for the self-renewal of LSCs and development of leukemia. METTL14 was found highly expressed in AML cells carrying t(11q23), t(15;17), or t(8;21), and loss of METTL14 significantly inhibits the proliferation and viability of these cells. Besides AML, high expression of METTL14 was also monitored in ALL and CML [125]. However, further explorations are requisite to understand its regulatory role in leukemogenesis and exploit new therapeutic targets.

#### WTAP

Up to now, it remains elusive whether WTAP participates in normal hematopoiesis and LSC self-renewal, but its association with leukemogenesis has been solidified. Several studies reported the highly expression of WTAP in AML, which is related with poor prognosis. Knockdown of WTAP inhibits proliferation and facilitates differentiation, suggesting that WTAP was an oncogene in AML [105, 106]. Further experiments revealed that WTAP could not execute its oncogenic role without METTL3, as WTAP failed to increase the proliferation level of leukemia cells with depressed METTL3 [130]. Particularly, WTAP plays a significant role in chemoresistance. In mechanism, deficiency in WTAP reduces the degradation of MYC mRNA in AML cells via a m6A-dependent manner [106]. WTAP knockdown increased sensitivity to chemotherapy drug daunorubicin of leukemia cells [105], while upregulation of WTAP promoted resistance to etoposide in AML [106]. Moreover, Hsp90 could prevent the degradation of WTAP in ubiquitin-proteasome pathway [106]. Shao et al. revealed the positive correlation between the WTAP and HIF1a, indicating that HIF1a may participate in the regulation of WTAP [131].

#### FTO

Distinct from methyltransferases, the demethylase FTO seems to play a minor role in normal HSCs [108]. However, FTO is engaged in the tumorigenesis and development of leukemia as an oncogene (Fig. 3). Inhibition of FTO attenuates LSC/LIC self-renewal by decreasing the expression of MYC and CEBPA [110]. On the contrary, the overexpression of FTO induces proliferation, curbs differentiation and apoptosis of AML cells, and promotes leukemogenesis in mice, highlighting its oncogenic role in AML [107]. High expression of FTO is found in specific AML subtypes by analyzing the whole genome of AML patients. In terms of the mechanism, FTO reduces expression of tumor suppressors RARA and ASB2 by impairing their mRNA stability, and thus facilitates the occurrence of leukemia [132]. Furthermore, overexpression of FTO contributes to TKI-resistance in leukemia cells via stabilizing MERTK and BCL2 mRNA. Thus, the combination of FTO inhibitors and TKIs could exhibit more favorable efficacy than TKI monotherapy for treating leukemia in mouse model [109].

These findings ignited scientists’ enthusiasm to explore therapeutic drugs targeting FTO. Rhein, the first naturally occurred FTO inhibitor, displays therapeutic efficacy in leukemia mice by competitively binding to the catalytic site of FTO [133]. Huang et al. discovered the MA, a nonsteroidal anti-inflammatory drug, selectively inhibits the demethylase activity of FTO [134]. Further research proved that MA2, the ester form of MA, could suppress glioblastoma progression [135]. MA derivative FB23-2 is also identified as selective inhibitor of FTO, and performs anti-leukemia functions in mouse AML model [136]. Another FTO inhibitor, R-2HG, exerts antitumor effects in AML by destabilizing the MYC/CEBPA mRNA [89]. Particularly, it was showed that R-2HG could effectively suppress aerobic glycolysis. And the underlying mechanism is disturbing the FTO-mediated post-transcriptional upregulation of critical glycolytic gene, PFKP and LDHB [108]. Su et al. found two small molecule FTO inhibitors, CS1 (bisantrene) and CS2 (brequinara), Which dramatically attenuate leukemia stem/initiating cell self-renewal and reprogram immune response by suppressing expression of immune checkpoint genes, especially LILRB4 [136]. Recently, Bai et al. have screened several acrylonitrile derivatives as potential FTO inhibitors. And they found that chlorine atom was involved in the binding between these small molecules and FTO [137]. Saikosaponin D is identified as an FTO-targeted drug to impair AML cells proliferation, inducing apoptosis and cell cycle arrest both in vitro and vivo, particularly in TKIs-resistant cells [138]. Besides, Wu, D et al. discovered that let-7b-5p mimics could downregulate the expression of FTO and thus upregulate c-MYC level in AML line cells [139].

#### ALKBH5

Despite dispensable in normal hematopoiesis and HSC functions, ALKBH5 is critical for the development and maintenance of LSCs (Fig. 3) [112]. Knockdown of ALKHB5 reduces proliferation and induces apoptosis of LSCs, attenuates its leukemogenic function in mice. For the mechanism, ALKBH5 enhances the expression of AXL by preventing the YTHDF2-medaited mRNA decay, and AXL is a receptor tyrosine kinase (RTK) phosphorylating FLT3 to accelerate development of AML [111, 135]. Also, ALKBH5 can stabilize TACC3 mRNA, a vital oncogene in multiple tumors, to promote self-renewal of LSC/LIC [135]. Meanwhile, ALKBH5 can induce AML chemoresistance by activating PI3K, MAPK, NF-kB, and JAK/STAT pathways, while KDM4C modulates the expression of ALKBH5 via promoting chromatin accessibility and recruiting MYC and Pol II to the promotor [111, 140]. Moreover, ALKBH5 can upregulate the expression of USP1, which mediates the glucocorticoid-resistance in T-ALL patients and cells via interacting with Aurora B [141]. These findings reveal the significance of ALKBH5 in the pathogenic process of leukemia. The abundance of ALKBH5 is found in AML patients with a normal karyotype, inv. [15], t(11q23), and t(8;21) and supposed to be related with poor prognosis.

Collectively, with a minor influence on normal hematopoiesis, ALKBH5 is considered as a crucial therapeutic target for leukemia. The crystallographic and biochemical studies indicated that the ALKBH5 inhibitors may be smaller in size than those for FTO, due to its smaller active cavity [142]. Selberg et al. assessed the antiproliferative effects of ALKBH5 inhibitors and discovered two compounds with potent inhibition against the proliferation of three leukemia cell lines, demonstrating the value of ALKBH5 inhibitor as an anti-leukemia strategy [143]. Le Zhang at al found that bioactive peptides could inhibit AML progression by reducing the ALKBH5-mediated m6A demethylation of EIF4EBP1 and MLST8 mRNAs, downregulating these two genes at both the RNA and protein levels [144].

#### YTHDF2

**Fig. 4:**
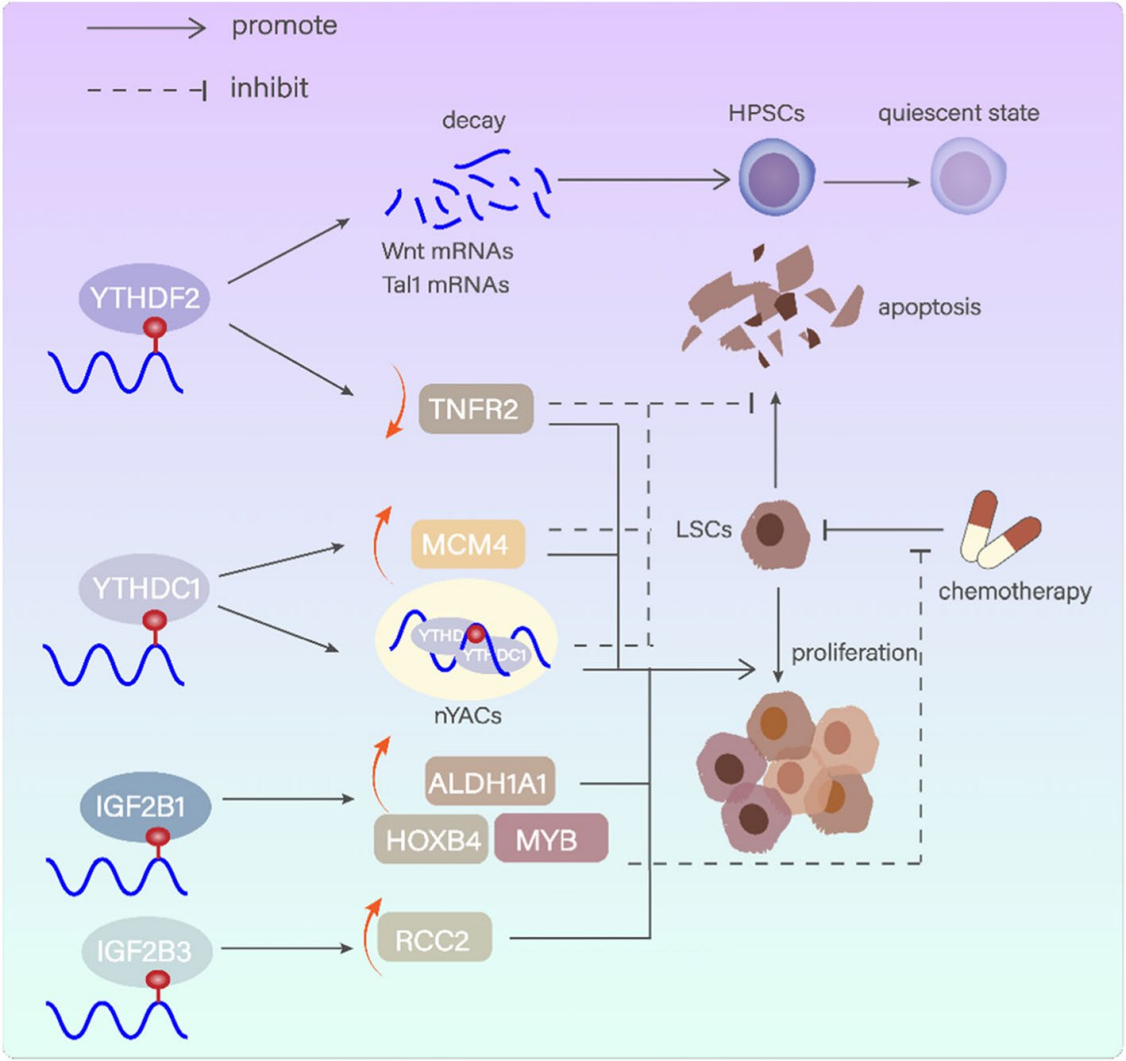
m6A readers in modulating normal and malignant hematopoiesis

It has been confirmed that YTHDF2 is crucial for maintaining the quiescent stage of HSCs and the deletion of YTHDF2 induces HSCs expansion in umbilical cord blood of human and mouse (Fig. 4) [103, 113, 114]. YTHDF2-deficient HSCs from young mice fail upon serial transplantation and chronically activate proinflammatory pathways. Moreover, hematopoiesis-specific YTHDF2 deficiency gives rise to a progressive myeloid bias, including loss of lymphoid potential, HSC expansion, and failure to reconstitute multilineage hematopoiesis [145]. YTHDF2 suppresses HSC self-renewal by promoting the mRNA degradation of key transcription factors (e.g., Tal1), which are critical for stem cell self-renewal [145]. Also, it has been found that loss of YTHDF2 decreases the m6A-dependent degradation, and thus abnormally activates Wnt signaling which could enhance the HSCs regeneration [103]. Moreover, YTHDF2 also plays a vital role in the proliferation and functional integrity of LSCs. For instance, it could prevent LSCs from apoptosis via decreasing tumor necrosis factor (TNF) receptor 2 (TNFR2) [113]. YTHDF2-deficient AML cells display impaired proliferative ability, increased apoptosis rate and decreased engraftment capacity [113].

#### Other regulators

##### YTHDC1

The contributions of YTHDC1 in normal hematopoiesis and leukemogenesis have been illuminated (Fig. 4). Yue et al. revealed that YTHDC1 participates in maintenance of HSCs and self-renewal of LSCs. Knockdown of YTHDC1 notably suppressed the regeneration of LSCs and primary AML cells rather than HSCs. YTHDC1 regulates leukemogenesis via MCM4, a critical DNA replication regulator [115]. Yuanming et al. confirmed that YTHDC1 is essential in maintaining cell viability and undifferentiated form in AML cells. They proved that the formation of nuclear YTHDC1-m6A condensates (nYACs) mediated by LLPS enables YTHDC1 to protect some mRNAs, such as MYC mRNA, from being degraded by PAXT complex and exosome, thus facilitates the process of leukemia [146].

##### IGF2BPs

IGF2BPs is reported to be significant for myeloid leukemia cell survival in an m6A-dependent manner, by interacting with YBX1 and stabilizing m6A-tagged RNA (Fig. 4) [62]. IGF2BP1 is supposed to maintain the integral properties of LSC by upregulating a set of crucial regulators, including HOXB4, MYB and ALDH1A1 [116]. Loss of IGF2BP1 decreased tumorigenicity, induced apoptosis and differentiation, and increased sensitivity to chemotherapy of leukemia cells. Moreover, high expression of IGF2BP1 is correlative with poor prognosis in AML patients [116]. Recently, Nan Zhang proposed that IGF2BP3 is required in leukemogenesis by interacting with RCC2 mRNA. It is demonstrated that knockdown of IGF2BP3 decreases AML cell viability in vivo, while overexpression of IGF2BP3 promotes proliferation and tumorigenesis of AML [117].

##### RBM15

The expression of RBM15 during hematopoiesis and its suppressive effects on myeloid differentiation has been validated. The underlying mechanism may be that RBM15 stimulates Notch signaling through RBPJkappa to disturb differentiation [118]. Overexpression of PRMT1 in acute megakaryocytic leukemia cell lines can downregulate RBM15 protein level via promoting its methylation and ubiquitylation. Subsequently, the expressions of several megakaryopoiesis-related genes are enhanced, such as GATA1, RUNX1, TAL1 and c-MPL, which blocks the terminal differentiation of megakaryocyte then [147].

**Table 2:** small-molecule inhibitors of m6A regulators

Targeting	Compounds	Functions	Reference
METTL3 inhibitor	Compound 1 and 2	Suppress m6A modification, exerts anti-tumor effects in AML models	[148]
	STM2457	Suppress m6A modification, reduce AML growth and increase differentiation and apoptosis	[128]
	UZH2	Suppress m6A modification in vitro	[129]
	UZH1a	Suppress m6A modification in vitro	[127]
FTO inhibitor	Rhein	Competitively binds the FTO catalytic domain and disrupts FTO from binding m6A-modified RNAs.	[133]
	Meclofenamic acid (MA)	Competes with FTO binding for the m6A-containing RNA, increase m6A levels	[134]
	MA2	Binds FTO active surface, induces m6A methylation, inhibits glioblastoma progression in mice	[135]
	FB23-2	Reduces expression of c-Myc and CEBPA and promotes RARA and ASB2, represses leukemia cell proliferation, survival, and leukemia progression in mice	[136]
	R-2HG	Induces degradation of c-Myc and CEBPA, promotes leukemia cell apoptosis, and inhibits leukemia growth in mice.	[108]
	CS1 and CS2	Attenuate LSC self-renewal, reprogram immune response by reducing LILRB4, sensitize leukemia cells to T-cell cytotoxicity, and show potent anti-leukemic efficacy in mouse models	[136]
	acrylonitrile derivative 1a	Binding FTO with chlorine atom, inhibits proliferation of leukemia cells	[137]
	Saikosaponin D	Inhibites AML cells proliferation, induce apoptosis and cell cycle arrest both in vitro and vivo, particularly in TKIs-resistant cells	[138]
	let-7b-5p mimic	Downregulate the expression of FTO and upregulate c-MYC level in AML line cells	[139]
ALKBH5 inhibitor	Compound 1 and 2 Bioactive peptide	Reduces leukemia cell viability inhibit AML cell proliferation and promote apoptosis in vitro, and reduce tumor growth in vivo	[143, 144]

## Conclusion

Accumulated evidences show that m6A modification the has broad influences on normal and malignant hematopoiesis, primarily by affecting mRNA stability and translation efficiency at the posttranslational level. It plays an important role in maintaining the normal functions of HSCs, promoting the occurrence and development of leukemia. Researchers are inspired to excavate the clinical potentials of m6A regulators as diagnostic biomarkers and therapeutic targets. However, the underlying rationales still remain unclear and the clinical applications require further investigations.

There are still challenges about the mechanism: (1) although it has been demonstrated that m6A modification affect RNA life through various pathway, it remains unclear how these selective effects are determined in different cellular environment, m6A-regulated phase separation provides a novel perspective to understand the specific effects; (2) following METTL3, METTL6 is identified to exert both methyltranferase activity-dependent and -independent functions, which inspires us to explore these regulators at a deeper level; (3) researchers have found that some regulators selectively function in either normal or malignant hematopoiesis while seem to be dispensable in the other process, the underlying rationale needs more exploration; (4) similar to other tumors, the existing studies of hematological show a relative bias toward writers and erasers, more attention is expected to be given to readers. Besides, emphasis shall be given to the interaction between m6A methylation and other epigenetic modifications.

With regard to the clinical applications, many opportunities and challenges exist: (1) The current m6A modification inhibitors and activators must be examined comprehensively with preclinical and clinical trials before clinical utilization. Actually, it takes several years and high cost for an anticancer drug into clinic use. Few of the currently available FTO inhibitors are limited by its poor sensitivity and/or selectivity; (2) similarly, whether m6A and its regulators can be used as potential biomarkers for diagnosis and prognosis of hematologic tumors need to be investigated thoroughly; (3) the potential interplay between m6A and ncRNAs provides a wide scope for future researches to explore more therapeutic targets; (4) traditional medicines and natural products have convincing safety validated by generations, and they are reliable sources of novel chemical structures. Moreover, AI-assisted approaches greatly improve the efficiency in screening, assessing and optimizing potential compounds [149].

Overall, previous progresses in m6A modification open up new avenues for future exploration. Given the significant role of RNA m6A in hematological malignancies, it is worthwhile to explore the therapeutic potentials and clinical benefits by targeting m6A regulators. We are convinced that the pathological mechanisms of hematological malignancies will be better understood and more feasible therapeutic strategies targeting m6A modification will be provided.

## Data Availability

Not applicable.

## Abbreviations

3'UTRs: 3'untranslated regions
ADAM19: A disintegrin and metallopeptidase domain 19
ALDH1A1: Aldehyde dehydrogenase 1 family member A1
ALKBH5: alkB homolog 5
AML: Acute myeloid leukemia
ASB2: Ankyrin repeat and SOCS box containing 2
ATRA: All-trans-retinoic acid
AXL: AXL receptor tyrosine kinase
BCL2: B cell lymphoma 2
CCR4-NOT: Glucose-repressible alcohol dehydrogenase transcriptional effector
CEBPA: CCAAT enhancer binding protein alpha
CEBPZ: CCAAT enhancer binding protein zeta
DNMT3A: DNA methyltransferase 3 alpha
EHT: Endothelial-to-hematopoietic transition
eIF: Eukaryotic translation initiation factor
FMRP: fragile X mental retardation protein
FOXM1: Forkhead box protein M1
FTO: Fat mass and obesity-associated protein
HIF: Hypoxia-inducible factor
HNRNP: heterogeneous nuclear ribonucleoprotein
HOXB4: Homeobox B4
HSC: hematopoietic stem cells
IGF2BP: insulin-like growth factor 2 mRNA-binding protein
KDM4C: Lysine demethylase 4 C
LDHB: Lactate dehydrogenase B
LILRB4: Leukocyte immunoglobulin like receptor B4
LSC: leukemia stem cells
lncRNA: long noncoding RNA
m6A: N^6^-Methyladenosine
MA: Meclofenamic acid
MCM4: minichromosome maintenance proteins
METTL: Methyltransferase-like
MERTK: MER proto-oncogene, tyrosine kinase
MTC: methyltransferase complex
MYB: MYB proto-oncogene, transcription factor
MYC: MYC proto-oncogene, bHLH transcription factor
NXF1: nuclear RNA export factor 1
Notch1a: Notch receptor 1a
pre-mRNA: precursor mRNA
PES1: pescadillo ribosomal biogenesis factor 1
PFKP: Phosphofructokinase platelet
PRMT1: protein arginine methyltransferase 1
PRRC2A: proline rich coiled-coil 2 A
PTEN: Phosphatase and tensin homolog
R-2HG: R-2-hydroxyglutarate
RARA: Retinoic acid receptor alpha
RBM15: RNA-binding motif protein 1
SAM: S-Adenosyl methionine
SPI1: Spi-1 proto-oncogene
SRSF: Serine and arginine-rich splicing factor
TACC3: Transforming acidic coiled-coil containing protein 3
TKI: Tyrosine kinase inhibitor
VCRs: vertebrate-conserved regions
VIRMA: Vir-like m^6^A methyltransferase-associated
WTAP: Wilms tumor 1-associated protein
XIST: X-inactive specific transcript
YAP: yes-associated protein
YTH: YT521-B homology
YTHDC1/2: YTH domain-containing protein 1/2
YTHDF1/2/3: YTH domain family protein 1/2/3
ZC3H13: Zinc finger CCCH-type containing 13
ZCCHC4: zinc finger CCHC-type containing 4
ZFD: zinc finger domain
ZNF217: Zinc finger protein 217
